# Which user errors matter during HIV self-testing? A qualitative participant observation study of men who have sex with men (MSM) in China

**DOI:** 10.1186/s12889-018-6007-3

**Published:** 2018-09-10

**Authors:** Chongyi Wei, Li Yan, Jianjun Li, Xiaoyou Su, Sheri Lippman, Hongjing Yan

**Affiliations:** 1 Department of Social and Behavioral Health Sciences, Rutgers School of Public Health, 683 Hoes Lane West, Office 312, Piscataway, NJ 08854 USA; 2 Southeast University, Nanjing, China; 3 Jiangsu Provincial Center for Disease Control and Prevention, Nanjing, China; 4 Peking Union Medical College, Beijing, China; 5 Center for AIDS Prevention Studies, University of California – San Francisco, San Francisco, USA

**Keywords:** HIV, Self-testing, HIV testing, Men who have sex with men

## Abstract

**Background:**

The World Health Organization recommends HIV self-testing (HIVST) as an additional approach to HIV testing services. We aimed to assess to what extent HIVST was conducted correctly by Chinese men who have sex with men (MSM) and to identify user errors during the HIVST process in order to inform strategies to optimize its use and thus reduce the number of undiagnosed HIV infections.

**Methods:**

Between February and March 2017, participant observations were conducted with 27 MSM in an east coastal city in China. In the presence, but without the assistance or orientation, of a trained HIV testing counselor, participants conducted HIVST (either finger prick or oral fluid) according to manufacturers’ instructions. Errors were recorded on checklists during direct observation and double checked afterwards by reviewing video files of the observations.

**Results:**

Overall, 12 participants (44.4%) had invalid test results due to user errors. Just five (18.5%) did not make any errors during the entire HIVST process. Failure to follow all the steps based on manufactures’ instructions was a common problem for both finger prick and oral fluid self-testers. For finger prick users, most errors occurred during the stage of collecting the specimen. In contrast, oral fluid users made most errors during the stage of testing the collected specimen.

**Conclusions:**

Although we found that user errors were common among MSM administering HIVST, this should not deter or discourage routine implementation and scale-up of HIVST as strategies can be implemented to facilitate the correct use of HIVST.

**Trial registration:**

This study was a part of a clinical trial: ClinicalTrials.gov (#NCT02999243); Registration date: December 20, 2016.

**Electronic supplementary material:**

The online version of this article (10.1186/s12889-018-6007-3) contains supplementary material, which is available to authorized users.

## Background

HIV self-testing (HIVST), the process in which a person collects his/her own specimen (oral fluid or blood) and then performs an HIV test and interprets the results in private, is recommended by the World Health Organization as an additional approach to HIV testing services [1]. Review of evidence demonstrated that HIVST was highly acceptable to many users including key populations, more than doubled uptake of HIV testing among men who have sex with men (MSM), could result in identifying an equivalent or greater proportion of HIV-positive individuals, and could achieve acceptable sensitivity (80–100%) and specificity (95–100%) [2–5]. This timely recommendation can help attain the first of the United Nation’s 90–90-90 targets, as many individuals remain unaware of their HIV serostatus [6].

HIVST is particularly suitable for expanding HIV testing services among MSM, who are disproportionally affected by HIV, and do not always access facility-based testing in contexts where MSM-related stigma and discrimination are prevalent [2, 7–9]. MSM account for a third and rising proportion of new HIV infections in China, which is in part due to lack of regular testing [10, 11]. Although not officially endorsed by the Chinese health authorities, HIVST kits are readily accessible through some community-based organizations and can be easily purchased on the Internet. Several recent cross-sectional surveys of Chinese MSM reported that between 20 and 29% of participants had ever self-tested for HIV [12–14]. Furthermore, one study found that 59% of men who self-tested reported HIVST as their first HIV testing experience [12].

As substantial numbers of Chinese MSM are already using HIVST, it is important to ensure that the tests are used correctly to improve diagnostic accuracy, especially in an unregulated market where quality of HIVST kits can vary. In fact, a study reported that just 24% of Chinese MSM who ever self-tested said they were “very confident” in the accuracy of their test results and 73% perceived self-testing to be less accurate compared to facility-based testing [13]. Therefore, this participant observation study aimed to assess to what extent HIVST was conducted correctly by Chinese MSM and to identify common user errors during the HIVST process. Such findings can inform strategies to optimize HIVST use and thus reduce the number of undiagnosed HIV infections.

## Methods

Between February 25th and March 5th, 2017, participant observations were conducted with 27 MSM in an east coastal city in China. Purposive sampling was used to recruit a diverse group of participants in terms of age, education and history of HIV testing. Participants were recruited through referrals from two local community-based organizations (CBOs) which offer HIV prevention services to MSM. To be eligible, participants had to be born male, 18 years old or older, a local resident, speak either Mandarin or the local dialect, have had sex with another male in the past 12 months, and self-report being HIV-negative or unknown status.

Participant observations were conducted in a private room at the Provincial Center for Disease Control and Prevention (CDC). Two types of HIV self-testing kits, finger prick (AIJI Colloidal Cold, HIV-1/2, NewScen Coast Bio-Pharmaceutical Co., Ltd., Tianjin, China) and oral fluid (Aware HIV-1/2 OMT, Beijing Marr Bio-Pharmaceutical Co., Ltd., Beijing, China), were offered to participants where they could choose to use either one based on preference. In the presence, but without the assistance or orientation, of a trained HIV testing counselor, participants conducted self-testing procedures according to the manufacturers’ text- and pictorial-based instructions. During the direct observations, the counselor recorded errors made by the participants using two checklists (one for each type of HIVST. Please see Additional files 1 and 2 for the checklists). Each participant observation was also video recorded. A research assistant reviewed the videos afterwards and double checked to ensure that the user errors were correctly marked on the checklists. All participants conducted a second HIVST with the assistance of the counselor after completing the self-testing procedures on their own. Participants who tested preliminary positive were offered confirmatory testing on site. The provincial CDC followed up with diagnosed HIV-positive individuals for CD4 and viral load testing and referred them to the local designated hospital for treatment.

Participation in the study was anonymous and voluntary. An incentive of 100 RMB (~ 15 USD) was offered to compensate for participants’ time and effort. The study was approved by the University of California San Francisco, Rutgers University and Jiangsu Provincial CDC’s Institutional Review Boards.

## Results

A total of 27 eligible men participated in the study. A majority (63.0%) of participants were between the ages of 20 and 29. About three quarters either completed some college (33.3%) or had an educational level of college or above (40.7%). Most (88.9%) were single/never married. Over half (59.3%) self-identified as gay while a third (33.3%) self-identified as bisexual. About half (44.4%) reported that they had a main male sex partner at the time of this study. Just under a third (29.6%) reported condomless anal sex in the past 6 months. In terms of history of HIV testing, 48.1% had never been tested for HIV while 33.3% said they had conducted HIV self-testing before (Table 1).

**Table 1:** Socio-demographic and behavioral characteristics of MSM participants (*N* = 27)

	n (%)
Age	
18–19	1 (3.7%)
20–29	17 (63.0%)
30–40	9 (33.3%)
Educational level	
High school or below	7 (25.9%)
Some college	9 (33.3%)
College or above	11 (40.7%)
Marital status	
Single	24 (88.9%)
Married	1 (3.7%)
Divorced/separated	2 (7.4%)
Employment status	
Full-time	18 (66.7%)
Part-time	1 (3.7%)
Student	7 (25.9%)
Unemployed	1 (3.7%)
Sexual orientation	
Gay	16 (59.3%)
Bisexual	9 (33.3%)
Heterosexual	0 (0%)
Unsure	2 (7.4%)
Have a main male sex partner	
Yes	12 (44.4%)
No	15 (55.6%)
Condomless anal sex in the past 6 months	
Yes	8 (29.6%)
No	19 (70.4%)
Ever tested for HIV	
Yes	14 (51.9%)
No	13 (48.1%)
Ever self-tested for HIV	
Yes	9 (33.3%)
No	18 (66.7%)

Of the 27 participants, 15 (55.6%) chose to use finger prick self-testing while the rest (44.4%) preferred oral fluid (Table 2). Overall, 12 (44.4%) had invalid test results due to user errors but were all re-tested as HIV-negative. Just five (18.5%) did not make any errors during the entire HIV self-testing process. Two (7.4%) self-tested positive and were re-tested HIV-positive.

**Table 2:** Numbers of errors made by MSM participants during HIV self-testing process and results of self-testing and retest (*N* = 27)

HIV self-testing modality: Finger prick (*N* = 15)									
Participant #	Age	Education	HIV testing history	Numbers of errors made				Test result	Retest
				Preparing the test (*n* = 0–2)	Administering the test (*n* = 0–4)	Interpreting the result (*n* = 0–1)	Total (*n* = 0–7)		
1	18–29	Some college	Ever self-tested	2	2	0	4	Negative	Negative
2	18–29	≥ College	Ever self-tested	1	2	0	3	Negative	Negative
3	18–29	Some college	Ever self-tested	0	3	1	4	Invalid	Negative
4	30–40	Some college	Ever self-tested	1	1	0	2	Negative	Negative
5	18–29	≥ College	Ever self-tested	0	1	1	2	Invalid	Negative
6	30–40	Some college	Ever self-tested	1	2	1	4	Invalid	Negative
7	18–29	Some college	Ever self-tested	1	2	0	3	Negative	Negative
8	30–40	≥ College	Ever self-tested	1	1	0	2	Negative	Negative
9	18–29	≥ College	Ever tested^a^	0	0	0	0	Negative	Negative
10	18–29	≤ High school	Never tested	1	4	1	6	Invalid	Negative
11	18–29	≥ College	Never tested	1	2	0	3	Positive	Positive
12	30–40	≤ High school	Never tested	0	3	1	4	Invalid	Negative
13	18–29	≥ College	Never tested	0	3	0	3	Negative	Negative
14	18–29	≥ College	Never tested	0	3	0	3	Negative	Negative
15	18–29	≥ College	Never tested	0	2	1	3	Invalid	Negative
HIV self-testing modality: Oral fluid (*N* = 12)									
				Numbers of errors made					
				Preparing the test (*n* = 0–3)	Conducting the test (*n* = 0–7)	Interpreting the result (*n* = 0–2)	Total (*n* = 0–12)		
16	18–29	≤ High school	Ever self-tested	3	6	2	11	Invalid	Negative
17	30–40	≤ High school	Ever tested^a^	1	4	0	5	Negative	Negative
18	18–29	Some college	Ever tested^a^	1	7	2	10	Invalid	Negative
19	18–29	≥ College	Ever tested^a^	0	2	2	4	Invalid	Negative
20	30–40	≥ College	Ever tested^a^	0	0	0	0	Negative	Negative
21	30–40	≤ High school	Never tested	1	6	2	9	Invalid	Negative
22	18–29	Some college	Never tested	0	0	0	0	Negative	Negative
23	18–29	≤ High school	Never tested	1	6	2	9	Invalid	Negative
34	18–29	≥ College	Never tested	0	0	0	0	Negative	Negative
25	30–40	Some college	Never tested	0	0	0	0	Negative	Negative
26	30–40	≤ High school	Never tested	0	4	2	6	Positive	Positive
27	18–29	Some college	Never tested	0	3	1	4	Invalid	Negative

^a^At a facility (e.g., CDC, CBO, hospital), excluding self-testing

Among finger prick self-testers, 6 (40.0%) had invalid test results (Table 2). Only one participant (6.7%) did not make any error, who was a frequent HIV tester although never self-tested before. Among those who made errors, total numbers of errors ranged from two to six (maximum = 7). During the stage of administering the test, a majority (10/14, 71.4%) made two or three errors (maximum = 4). The most common errors included: difficulties in using the lancet and the micro pipette to draw blood; insufficient amount of blood drawn from finger; and failure to follow all the steps. Notably, of the 8 participants who self-tested before, three (37.5%) had invalid test results and all made some errors during the process.

Among oral fluid self-testers, 6 (50.0%) had invalid test results (Table 2). Four participants (33.3%) did not make any error. Among those who made errors, total numbers of errors ranged from four to 11 (maximum = 12). During the stage of administering the test, a majority (6/8, 75.0%) made four or more errors (maximum = 7). The most common errors included: failure to follow all the steps; removing the membrane surface from the assay test strip; and placing the test strip upside down in the tube containing the diluted specimen.

## Discussion

This participant observation study found that when unassisted, a majority of Chinese MSM participants made some user errors during the process of conducting HIVST. Some of the errors were significant enough that almost half of the test results were rendered invalid. Our finding is consistent with prior research that sensitivity of HIVST was reduced among individuals in unsupervised or unassisted settings [5, 15]. Failure to follow all the steps according to manufactures’ instructions was a common problem for both finger prick and oral fluid self-testers. For finger prick users, most errors occurred during the stage of collecting the specimen. In contrast, oral fluid users made most errors during the stage of testing the collected specimen.

There were no clearly discernible patterns between participants’ histories of HIV testing and numbers of user errors or self-testing results; having previously tested for HIV did not facilitate the conduct of HIVST. In fact, a third of the invalid test results were observed among participants who self-tested before. It did appear, however, that participants with lower educational level were more prone to making errors. Of the 7 men who completed high school or below, almost three quarters 9 (5/7, 71.4%) had invalid test results. This may not be surprising given that the manufactures’ instructions were mainly text-based.

Several limitations of this study should be noted. First, our sample of participants were referred by CBOs that provided HIV testing services to MSM. However, we purposefully recruited men who were diverse in terms of age, education, and history of HIV testing. Second, we only provided HIVST kits from two manufacturers. Kits from other manufacturers might have included improved instructions. However, these two manufactures are currently the main vendors of HIVST kits in China. Third, due to the presence of an HIV testing counselor and video recording, some participants might have felt uneasy or nervous leading them to make unnecessary user errors. However, our finding is consistent with those from previous studies [5, 15]. Finally, this was a relatively small sample of MSM participants, but the qualitative methodology (i.e., participant observation) provides contextual information of the HIVST process and is not intended to generalize. The findings could inform future surveys that quantify the error types and their magnitude.

## Conclusions

Although we found that user errors were common among MSM administering HIVST, this should not deter or discourage routine implementation and scale-up of HIVST as strong evidence shows that the benefits of reaching untested MSM and increasing frequency of testing among this population outweigh the disadvantages (e.g., reduced but acceptable sensitivity). A few strategies could be implemented to facilitate the correct conduct of HIVST. First, in addition to text- and pictorial-based instructions, manufacturers should provide video instructions that use simple but clear language (e.g., OraSure Technologies provides video instructions in multiple languages). This would especially benefit men with low or limited health literacy. Second, for those who wish to access free kits through CBOs, HIV testing counselors could offer orientation or instruction before men take the kits home. Finally, health authorities should continuously and comprehensively monitor the quality of HIVST kits sold on the market so that individuals can make informed choices.

## Additional files

Additional file 1: Participant observation checklist: checklist for finger prick HIV self-test. (DOC 16 kb)

Additional file 2: Participant observation checklist: checklist for oral swap HIV self-test. (DOC 28 kb)

## Abbreviations

CBOs: Community-based organizations
CDC: Center for Disease Control and Prevention
HIVST: HIV self-testing
MSM: men who have sex with men

## References

[1] World Health Organization (2016). Guidelines on HIV self-testing and partner notification: supplement to consolidated guidelines on HIV testing services.

[2] Figueroa C, Johnson C, Verster A (2015). Attitudes and acceptability on HIV self-testing among key populations: a literature review. AIDS Behav.

[3] Katz D, Golden M, Hughes J, et al. HIV self-testing increases HIV testing frequency among high-risk men who have sex with men: a randomized controlled trial. Presented at: 8^th^ international AIDS society conference; 19–22 July 2015; Vancouver, Canada; 2015.

[4] Jamil M, Prestage G, Fairley C, et al. Access to HIV self-testing doubles the frequency of HIV testing among gay and bisexual men at higher risk of infection: a randomised controlled trial. Presented at: 21st international AIDS conference; 18-22 July; Durban, South Africa; 2016.

[5] Pant Pai N, Sharma J, Shivkumar S (2013). Supervised and unsupervised self-testing for HIV in high- and low-risk populations: a systematic review. PLoS Med.

[6] UNAIDS. Fast-Track – Ending the AIDS epidemic by 2030. Geneva: Joint United Nations Programme on HIV/AIDS; 2014.

[7] Beyer C, Baral SD, van Griensven F (2012). Global epidemiology of HIV infection in men who have sex with men. Lancet.

[8] Sullivan PS, Carballo-Dieguez A, Coates T (2012). Successes and challenges of HIV prevention in men who have sex with men. Lancet.

[9] Beyer C, Baral SD, Collins C (2016). The global response to HIV in men who have sex with men. Lancet.

[10] China Ministry of Health, UNAIDS, WHO (2011). Estimates for the HIV/AIDS epidemic in China.

[11] Zou H, Hu N, Xin Q (2012). Beck J. HIV testing among men who have sex with men in China: a systematic review and meta-analysis. AIDS Behav.

[12] Han L, Bien C, Wei C (2014). HIV self-testing among online MSM in China: implications for expanding HIV testing among key populations. J Acquir Immune Defic Syndr.

[13] Yan H, Yang H, Raymond HF (2015). Experiences and correlates of HIV self-testing among men who have sex with men in Jiangsu Province. China AIDS Behav.

[14] Qin Y, Tang W, Nowacki A (2017). Benefits and potential harms of HIV self-testing among men who have sex with men in China: an implementation perspective. Sex Transm Dis.

[15] OraSure Technologies (2012). Final Advisory Committee Briefing Materials: Available for Public Resease. OraQuick® In-Home HIV Test.

