# Supplementary material for: Which user errors matter during HIV self-testing? A qualitative participant observation study of men who have sex with men (MSM) in China
Source: BMC Public Health. 2018 Sep 10;18:1108. doi: 10.1186/s12889-018-6007-3 (PMC6131779; doi:10.1186/s12889-018-6007-3)
Supplement: Supplementary file 1 — Participant observation checklist: checklist for finger prick HIV self-test. (DOC 16 kb) [file 12889_2018_6007_MOESM1_ESM.doc]

**HIV-1/2 antibody finger test checklist**

**Methods: whole blood test**

1. Read the instructions of the test kit carefully

True □ False □

2. Tear open a test kit, and put the reagent on a test-bed horizontal.

True □ False □

3. Press the end of the left ring finger and clean the finger using alcohol prep pad

True □ False □

4. Place the Lancet at the finger and press the trigger to prick the finger

True □ False □

5. Collect 1-2 drops of the blood sample (25-35 ml) using the micro pippette and place the sample into the Sample Well (S), DO NOT drop the finger blood into the Sample Well (S) directly.

True □ False □

6. Bandage the wound using BAND-AID.

True □ False □

7. Read the results within 15-30 minutes

True □ False □

**Interpretation of result:**

1. Positive result: two line appear. If both a test and control line appear , i.e. two lines appear on the test strip, in the Test Zone and Control Zone, respectively, the result is considered positive. One of these lines maybe darker than the other. Do NOT panic and please confirm the result from the local CDC.

2. Negative result: only control line appears on the test strip in the control zone.

3. Invalid: there is no control line in the Control Zone. It means the device may be faulty, please test again using a new kit.
