# Supplementary material for: Which user errors matter during HIV self-testing? A qualitative participant observation study of men who have sex with men (MSM) in China
Source: BMC Public Health. 2018 Sep 10;18:1108. doi: 10.1186/s12889-018-6007-3 (PMC6131779; doi:10.1186/s12889-018-6007-3)
Supplement: Supplementary file 2 — Participant observation checklist: checklist for oral swap HIV self-test. (DOC 28 kb) [file 12889_2018_6007_MOESM2_ESM.doc]

**Operating instructions of the Aware HIV-1/2 Oral Mucosal Transudate (OMT) test for detection of antibodies**

1. The OMT is intended to be used as an aid of voluntary counseling and testing of HIV diagnosis.

2. To protect your own privacy, please use the OMT kit in a safe place.

3. The remained HIV-positive sperms in mouth might affect the test results, while dental plaque, periodontitis, and periodontal disease will not affect the test results.

4. This test should be performed at ambient temperature (15-30℃)

5. The oral specimen can only be collected 30 minutes after teeth brushing and gargling. Do not retest sooner than 30 minutes after a previous collection.

6. According the Calypte experiment, the test results will not be affected by smoking, drinking alcohol, coffee, milk, tea, carbonated beverage, juice, coke, water and eating. Please perform the test after 10 minutes of having above behaviors.

7. Remove the clean Collection Swab from the pouch and avoid touching the cloth end of the swab.

8. Try to insert the swab into the back corner of the gum line in the mouth, brush the entire gum line up and down with the cloth end of the swab.

9. Open a foil pouch and remove the assay test strip. Avoid touching the membrane surface in the middle of the strip and water absorption zone with your fingers.

10. Place the assay test strip in the tube containing the diluted specimen and wait 30 minutes. Read the test results against a background with adequate lighting. If two lines appear, the result can be interpreted as positive.

**Correct operation:**

1. Read the instructions of the kit carefully.

True □ False □

2. Preparation

A. Insert the tube to the sample well and remove the cap, turn the package upside down.

True □ False □

B. Turn the tube containing sample and buffer upside down 3 times, and insert it on the sample well of the kit.

True □ False □

C. Turn the tube left and right and remove the cap.

True □ False □

★ Operate carefully and avoid splashing when insert the swab into the liquid in the open tube of sample buffer. Once splashing, take it easy since there is no harmful substance in the the liquid.

3. Specimen Collection

Please use the swab to collect the specimen

1. Brush the upper gum line up and down using one side of the swab (5-6 seconds)

True □ False □

B. Brush the lower gum line using the other side of the swab (5-6 seconds)

True □ False □

★ Specimen Collection is of most importance for a valid test.

Gum line refers the junction area between the gum and the inner lip. Apply moderate pressure to brush the entire gum line using the swab until reaching the other corner of the mouth. we collect the Oral Mucosal Transudate, rather than saliva.

★ Oral Mucosal Transudate is a fluid that passes through a mucous membrane lining between the teeth and the gum inside of the mouth. It is one of the serum transudates and a component of the whole saliva.

4. Mixing up the swab specimen and the buffer

A. Insert the swab into the liquid in the open tube of the sample buffer.

True □ False □

B. Rub both sides of the swab against the sides of the tube to completely mix the sample and the buffer.

True □ False □

★ Plunge the swab up and down 7-8 times in the sample buffer tube

C. Wring out the fluid as the swab is being removed, to keep the specimen in the tube as much as possible.

True □ False □

D. Discard swab

True □ False □

5. Testing

Insert the assay test strip into the tube to test the sample

A. Open a foil pouch and remove the Aware HIV-1/2 OMT test strip.

True □ False □

B. Place the assay test strip in the tube containing the diluted specimen, with the arrows on the assay test strip pointing down.

True □ False □

★ wait 20 minutes

Avoid touching the membrane surface in the middle of the strip with your fingers.

6. Reading the result after 20 minutes and do not read the result more than 45 minutes after beginning the test.

A. Remove the test strip and compare with the picture at the kit bottom.

True □ False □

B. If a single line appears on the test strip in the Control Zone, the result is negative. It suggests the absence of reactive antibodies in the specimen.

True □ False □

**Interpretation of result:**

1. Positive result: two line appear. If both a test and control line appear , i.e. two lines appear on the test strip, in the Test Zone and Control Zone, respectively, the result is considered positive. One of these lines maybe darker than the other. Do NOT panic and please confirm the result from the local CDC.

2. Negative result: only control line appears on the test strip in the control zone.

3. Invalid test: there is no control line in the Control Zone. It suggests the device may be faulty, please test again using a new kit.
